# Correction: A fluorescent biosensor for cardiac biomarker myoglobin detection based on carbon dots and deoxyribonuclease I-aided target recycling signal amplification

**DOI:** 10.1039/d2ra90047e

**Published:** 2022-04-27

**Authors:** Jishun Chen, Fengying Ran, Qinhua Chen, Dan Luo, Weidong Ma, Tuo Han, Ceming Wang, Congxia Wang

**Affiliations:** Department of Cardiology, The Second Affiliated Hospital of Xi’an Jiaotong University Xi’an Shanxi 710004 China wcx622@163.com +86-02987679770; Affiliated Dongfeng Hospital, Hubei University of Medicine Shiyan Hubei 442008 China; Shennongjia Golden Monkey Key Laboratory of Conservation Biology in Hubei Province Shennongjia Hubei 442400 China

## Abstract

Correction for ‘A fluorescent biosensor for cardiac biomarker myoglobin detection based on carbon dots and deoxyribonuclease I-aided target recycling signal amplification’ by Jishun Chen *et al.*, *RSC Adv.*, 2019, **9**, 4463–4468, https://doi.org/10.1039/C8RA09459D.

The authors are publishing this correction to draw the reader’s attention to three related papers that should have been cited as ref. 43–45 in this *RSC Advances* article. These have been given below as ref. 1–3, respectively.

On page 4464, a citation to ref. 43 should be added to the end of the sentence beginning “Ultrapure water…”. Therefore, the sentence should be changed to “Ultrapure water obtained from a Millipore water purification system (18.2 MΩ cm resistivity, Milli-Q Direct 8) was used in all runs.^43^”

On page 4465, citations to ref. 43 and 44 should be added at the end of the sentence beginning “The fluorescence intensity…”. Therefore, the sentence should be changed to “The fluorescence intensity and the value of *F*_1_/*F*_0_ are selected to evaluate the effects of the reaction conditions on the sensing performance of the assay.^43,44^”

On page 4467, a citation to ref. 45 should be added to the end of the sentence beginning “Aptamer can recognize…”. Therefore, the sentence should be changed to “Aptamer can recognize MB specifically, and thus provide a promising method for its assay, this promising strategy might prove feasible as an MB assay for real samples.^45^”

The authors also regret that there are portions of unattributed text overlap in the Results and discussion and Conclusion sections with other papers, including ref. 43–45.

The Royal Society of Chemistry apologises for these errors and any consequent inconvenience to authors and readers.

## Supplementary Material
